# MarkerScan: Separation and assembly of cobionts sequenced alongside target species in biodiversity genomics projects

**DOI:** 10.12688/wellcomeopenres.20730.1

**Published:** 2024-02-13

**Authors:** Emmelien Vancaester, Mark L. Blaxter

**Affiliations:** 1 Tree of Life, Wellcome Sanger Institute, Hinxton, England, UK

**Keywords:** bioinformatics tools, cobionts, genome sequencing, database contamination, eukaryotic genomics

## Abstract

Contamination of public databases by mislabelled sequences has been highlighted for many years and the avalanche of novel sequencing data now being deposited has the potential to make databases difficult to use effectively. It is therefore crucial that sequencing projects and database curators perform pre-submission checks to remove obvious contamination and avoid propagating erroneous taxonomic relationships. However, it is important also to recognise that biological contamination of a target sample with unexpected species’ DNA can also lead to the discovery of fascinating biological phenomena through the identification of environmental organisms or endosymbionts.

Here, we present a novel, integrated method for detection and generation of high-quality genomes of all non-target genomes co-sequenced in eukaryotic genome sequencing projects. After performing taxonomic profiling of an assembly from the raw data, and leveraging the identity of small rRNA sequences discovered therein as markers, a targeted classification approach retrieves and assembles high-quality genomes. The genomes of these cobionts are then not only removed from the target species’ genome but also available for further interrogation.

Source code is available from https://github.com/CobiontID/MarkerScan. MarkerScan is written in Python and is deployed as a Docker container.

## Plain Language Summary

This article addresses a common issue in genetic research: the accidental mixing of genetic information from different species in public databases, often due to mislabelling or contamination. Interestingly, this ‘contamination’ can sometimes lead to exciting discoveries, like identifying DNA from unexpected species in a sample, revealing insights about organisms that live in the environment of the target organism.

In our study, we developed a tool called MarkerScan for identifying these additional species found alongside the target species in eukaryotic genome sequencing projects. The method includes a way to sequence the whole genomes of the additional species. Our method involves sorting through the genetic data to identify certain small RNA sequences, which we then use as markers. These markers help to classify and assemble high-quality genomes from these additional species. This not only cleans up the main target species’ genome data but also provides new, valuable genomes for further exploration.

## Introduction

With advances in sequencing technology and the launch of several large-scale biodiversity genomics projects ^1– 3^, the total number of nucleotide bases in public repositories is doubling around every 18 months ^4^. The issue of contamination in public databases, where DNA or RNA from a contaminating species is tagged as coming from the target species, is a pressing concern ^5^, exacerbated by the constant influx of new sequencing data. It is therefore crucial that assemblies are subjected to pre-submission checks to confirm the allocation of sequence data to the intended target species and to avoid adding yet more confusion to the public databases.

Although contamination can be introduced during many stages of handling of the sample, eukaryotic species often accommodate symbionts or can be part of complex stable communities. These types of relationships can range from parasitic to commensal to mutually beneficial, and can either be facultative or obligate interactions. Insects often harbour endosymbionts, such as *Spiroplasma*, *Wolbachia* and *Sodalis*, while legumes depend on *Rhizobium* for nitrogen fixation in their nodules. Mutualistic partners can protect the host from viruses or parasites ^6^, can aid through nutrient supplementation ^7, 8^ and even establish magnetoreception ^9^. Moreover, these close connections can influence the host genome as it can lead to gene loss due to metabolic interdependency and close proximity can lead to increased rates of horizontal gene transfer ^10, 11^. Sequencing of DNA extracted from whole organisms effectively samples their local ecosystems including environmental species, food items and infections. Data generated from whole animals will also sequence components of the gut microbiome, and the phyllosphere and rhizosphere microbial communities will be sampled when sequencing from whole plants. These non-target genomes hold keys to the ecology of the target species, and simply discarding them will lose this information. Because we do not know *a priori* the biological relationships between the non-target organisms and the target species, we define them as ‘cobionts’. Therefore, genome projects should aim to reconstruct the genomes of all species present in a sample, treating single-species samples as low complexity metagenomes.

It is possible to perform taxonomic classification of data from primary assemblies. Taxonomic sequence classification can be performed in either a reference-free, which only uses intrinsic features or a reference-dependent manner, which exploits prior sequence information. Database-free methods often use coverage and compositional sequence patterns, such as tetranucleotide frequency spectra, to perform clustering of a primary assembly ( Table 1). This binning approach is heavily used in metagenomics due to the high gene density and largely consistent sequence patterns observed within bacterial and archaeal genomes. However, the often large repetitive content and non-coding fraction of eukaryotic genomes makes them less amenable to these methods, and effective binning of mixed prokaryotic and eukaryotic data remains challenging. Bins generated by coverage and compositional patterns must be taxonomically classified *post hoc*, and another suite of methods have been developed to perform taxonomic profiling, such as GTDK-Tk ^12^, MetaPhlAn4 ^13^, MEGAN-LR ^14^ and Centrifuge ^15^.

**Table 1.** Non-exhaustive overview of methods aiming to partition species in sequence data sets.

Methodology	Tool	Sequence attributes	Clustering method
Metagenomic binning tools	MetaBat2 ^19^	4-mer & coverage	Graph-based clustering
	MaxBin2 ^20^	4-mer & coverage	Expectation-maximisation
	Concoct ^21^	4-mer & coverage	Gaussian mixture models
	MetaBCC-LR ^35^	3-mer & coverage Dimensionality reduction	DBSCAN
	VAMB ^36^	4-mer & coverage Variational autoencoder	Iterative medoid clustering
	GraphMB ^37^	4-mer & coverage & graph Variational autoencoder	Iterative medoid clustering
Classification tools	BlobToolKit (BTK) ^5^	Visualisation method of (k=1) GC & coverage	
	Conterminator ^17^	Database of hashed 24-mers, followed by MMMSeqs2 alignment	
	Kraken2 ^18^	Database of hashed 35-mers, followed by last common ancestor algorithm	
	FCS-GX ^22^	Database of hashed 38-mers, followed by 20-mer search	

Alternatively, each sequence can be compared to a trusted database to detect its most similar sequence. Historically, BLAST ^16^ has been heavily used as a search tool. However, the huge expansion of sequence information means that exhaustive approaches are no longer feasible on a large scale. Databases of hashed fixed length sequence fragments ( *k*-mers) of known origins can be used to classify query sequences and reduce the search space and thus speed up the more traditional alignment phase (e.g. Conterminator ^17^) or can be used in a last common ancestor computation of the best-matching taxa (e.g. Kraken2 ^18^) ( Table 1). However, how well these approaches succeed in classifying the query ultimately depends on how densely the sequence space is represented within the limits of sequence divergence.

We have built a new workflow, MarkerScan, that leverages assemblies that can be generated from the intrinsic high quality of accurate, long-read data and deploys an assemble-identify-gather-reassemble process to deliver high-quality cobiont assemblies from data generated to assemble a eukaryotic target species’ genome. While current methods perform taxonomic classification either after partitioning of the sequence data in case of metagenomic binners (e.g. MetaBat2 ^19^, MaxBin2 ^20^, Concoct ^21^) or as part of the classification process (e.g. Kraken2 ^18^, Conterminator ^17^, FCS-GX ^22^), MarkerScan differentiates itself by implementing the taxonomic screening prior to classification. While some taxonomic classifiers rely on a whole range of protein markers ^12^, MarkerScan uses the universal marker small subunit rRNA (SSU; also known as 18S rRNA or 16S rRNA) to attribute family-level assignment to each detected ribosomal gene region. The SSU locus has been one of the most used, in both metabarcoding and metagenomics, to place sampled diversity in a global phylogeny ^23^. SSU is universal because of its essentiality and is orthologous between all taxa, but importantly has variation sufficient to discriminate most taxa (except within some species groups ^24^). Well-populated databases are available linking SSU sequences to taxonomy, such as SILVA ^25^, RDP ^26^, GreenGenes ^27^ and EukRef ^28^. Accurate, long-read sequencing now enables the direct read-out of SSU loci from raw data and direct classification of the SSU sequences discovered allows fast taxonomic classification of the likely contributors to a sequence set ^29– 32^.

In the context of reference genome sequence generation, current practice focuses on the identification and removal of any sequences not belonging to the host. MarkerScan aims to change this approach by additionally generating high quality genomes for all additional species present in the sample. MarkerScan does this by first identifying the taxa likely to be present in a sample through classification of the SSU genes present, uses targeted compositional binning for each family of organisms identified using Kraken2 ^18^, and reassembles within each bin to produce cobiont assemblies that are assessed using BUSCO ^33^ and nucleotide similarity ^34^.

## Methods

### Workflow methodology

**Figure 1.**
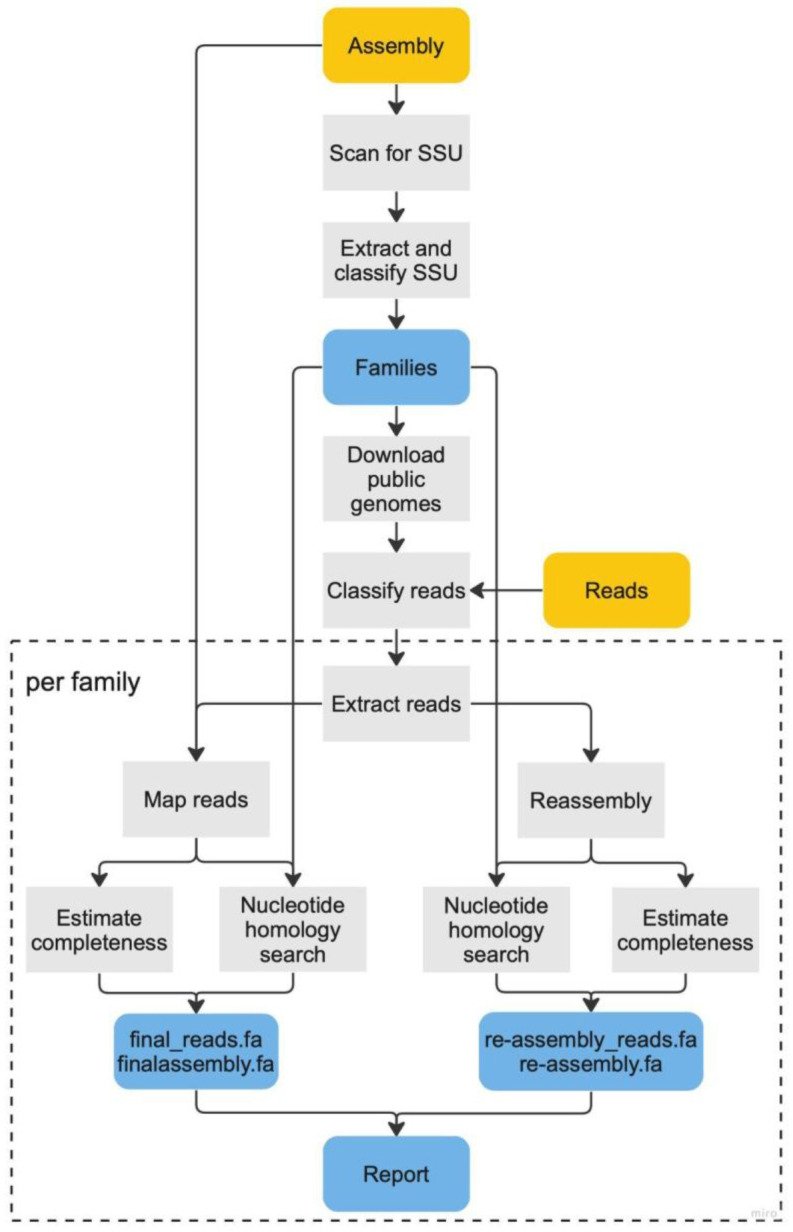
MarkerScan workflow schematic. Workflow describing the main steps of identifying and reassembling additional components in a host assembly using MarkerScan. Yellow boxes signify input datasets, blue boxes are results, while grey boxes represent intermediate steps. After detecting all families present in the assembly based on the taxonomic classification of the small subunit rRNA, a custom database is created to classify all reads with Kraken2. Reads are classified and assembled for each family bin, and the quality of the corresponding assembly/assemblies is/are assessed and compared to their reassembly.

We envisage a use case of the assembly of a target eukaryotic species’ genome using PacBio HiFi accurate long reads, as is standard in the Darwin Tree of Life project (DToL) ^3^ and other Earth BioGenome Project biodiversity genomics initiatives ^1, 2^. To separate additional species within the assembly of the target species, we developed a workflow called MarkerScan, using the workflow manager Snakemake (version 5.23.0) ^38^. A schematic representation of the full pipeline is presented in Figure 1. MarkerScan uses a set of reference databases, which are automatically downloaded when absent or more than one month old. These databases include SILVA (current version 138.1), NCBI Taxonomy and RefSeq Organelles. To run the workflow the user is required to generate a configuration file which points to both the location of an (unfiltered) assembly in FASTA format and PacBio HiFi read file in either compressed FASTQ or FASTA format. The user also needs to provide the full scientific name of the host species as available in NCBI taxonomy. An example of these inputs is given in supplementary file 1 as well as on the Github page.

First, the species composition of the sample is assessed by determining the presence of small subunit rRNA (SSU) regions in the assembly. An HMM profile was generated representing the archaeal, bacterial, eukaryotic and microsporidian SSU alignments (RF00177, RF01959, RF01960, RF02542) taken from RFAM ^39^ and is used to screen an assembly with nhmmscan from the HMMER Suite (version 3.3.1) ^40^. Hits with an e-value <10 ^−150^ or an aligned length of >1,000 nucleotides are retained. The putative SSU sequences are extracted and redundancy of the SSU set is reduced by collapsing any that have more than 99% identity using CD-HIT (version 4.8.1) ^41^. The SSU loci are classified by comparison to the SILVA SSU database (version 138.1) ^25^ using sina (version 1.7.0) ^42^. Only matches having higher than 90% nucleotide identity are retained to derive a taxonomic classification to family level of each query SSU region. We use a consensus rule of 80% concordance among the top 20 best hits using both the NCBI ^43^ and SILVA taxonomies ^44^. From this process MarkerScan generates a catalogue of the likely distinct cobionts present in the data, and their assignment to family.

For every observed family, all publicly available genomes are downloaded using NCBI Datasets ^45^. The genomes of the closest relatives of the host species are also downloaded. After masking using dustmasker ^46^, these genomes are transformed into a *k*-mer database ( *k* = 50) compatible with Kraken2 (version 2.0.7) ^18^. All the raw sequencing reads are classified against this database using Kraken. The family taxonomic level was chosen as it provides a balance between generation of a *k*-mer database that contains sufficient representation of the sequence diversity of the likely genome being sought, as well as being stringent enough to reduce false positive classification.

The classified reads are used in two ways. Eukaryotic genomes can contain horizontally acquired sequence from prokaryotic and eukaryotic symbionts and pathogens, especially when the association between host and symbiont is long term. To avoid inadvertently classifying nuclear insertions as part of a microbial genome, only contigs fully covered by the classified reads were retained (using minimap2, version 2.17, and settings “-x map-hifi”) ^47^. The best BUSCO lineage (version 5.2.2) ^33^ was chosen based on the family classification of the bin and the completeness of the contig set was assessed. Finally, contigs are retained only if they contain a marker gene as proposed by BUSCO or have sufficient sequence similarity according to nucmer (mummer4, version 4.0.0rc1) ^34^ to the downloaded public genomes of family members. In addition, the binned reads are reassembled using hifiasm (version 0.14) ^48^. Contigs generated in the reassembly are also only retained if they either contain a BUSCO marker gene or have sufficient sequence similarity according to nucmer (mummer4, version 4.0.0rc1) ^34^ to the downloaded public genomes of family members.

### Mock community analyses

To assess the classification accuracy and precision of MarkerScan, it was compared to five other methods: MetaBat2 (version 2.15) ^19^, MaxBin2 (version 2.2.7) ^20^, Concoct (version 1.1.0) ^21^, BTK (version 2.6.4) ^5^ and FCS-GX (version 0.4.0) ^22^. Publicly available PacBio HiFi data from two mock communities (ZymoBIOMICS® Gut Microbiome Standard and ATCC MSA 1003 community ^49^), which have known species composition and stoichiometry and where reference genomes of the exact strain of each microbial component are available, were assessed ( Table 2). These communities were assembled with both metaFlye (version 2.8.3) ^50^ and hifiasm (version 0.13-r308) in meta mode (0.1-r022) ^29^ and compared to their respective reference genomes using minimap2 (-x asm5) (version 2.17) ^47^. Completeness of all bins was assessed using CheckM (version 1.2.2). F1 score was calculated by how many contigs were correctly classified normalised by their length with the formula: (2 × precision × recall)/(precision + recall).

**Table 2.** Reference datasets analysed in this study.

Mock communities					
*Dataset name*		*Taxonomic group*	*Expected genome * *size*	*Raw data accessions*	*Reference*
ATTC MSA-1003		20 bacteria	Total 62 Mb	SRR11606871	49
Zymobiomics Gut Microbiome Standard		2 fungi and 19 bacteria	Total 93 Mb	SRR13128014	49
Target species					
*Species name*	*Common * *name*	*Taxonomic group*	*Reference * *assembly ID and * *genome size*	*Raw data accessions*	*Reference*
*Apoderus coryli*	hazel-leaf roller weevil	Coleoptera, Arthropoda	GCA_911728435.2 428 Mb	ERR6412370	51
*Mythimna impura*	smoky wainscot	Lepidoptera, Arthropoda	GCA_905147345.3 935 Mb	ERR6590581, ERR6576317	52
*Thunnus albaceres*	yellowfin tuna	Osteichthyes, Chordata	GCA_914725855.1 792 Mb	ERR7012643, ERR7979904, ERR7012645, ERR7012644	53
*Cladonia squamosa*	dragon cup lichen	Ascomycota, Fungi (in photosymbiosis with Trebouxiales)	GCA_947623385.2 38 Mb	ERR9871430	3
*Chondrosia reniformis*	kidney sponge	Demospongiae, Porifera	GCA_947172415.1 117 Mb	ERR10224860	54

### Case study analyses

Five species of varying taxonomy, genome size and expected complexity of cobiome were analysed ( Table 2). For each, a primary contig assembly was generated with hifiasm (version 0.12-r304) ^48^ for PacBio Sequel IIe HiFi datasets from DToL and the Vertebrate Genomes Project (VGP). The degree of contamination in these assemblies was quantified with three different tools: FCS-GX (version 0.4.0) run with database version 1 (09/07/22), BlobToolKit (version 4.1.7) run with UniProt reference proteomes and nt databases (26/07/23) and MarkerScan. Contig assemblies were mapped to the final released, curated and cleaned chromosome-level assemblies of the target species ( Table 2) using minimap (-x asm5) (version 2.17) ^47^ to identify contigs corresponding to true host sequences, and tool-derived classifications as “false” when their assignments were to families other than that of the known species present in the sample.

## Results

### Evaluation of MarkerScan performance on gold standard data

To assess how well both reference-free and database-aware methods perform on sequence classification, six tools were compared in how successfully and correctly they binned data from two publicly available mock communities. The tools assessed were the reference-free binning methods Concoct, MaxBin2 and MetaBat2, which were assessed on their binning capabilities without taking taxonomic classification into account, and the taxonomic classification methods FCS-GX, BlobToolKit (BTK) and MarkerScan, for which we additionally correct identification at the family level. The mock communities represent low-complexity metagenomic datasets, composed out of fewer than twenty species and have a known species composition and abundance ^49^. The ATTC MSA-1003 mock community is composed of a total of twenty bacterial species with five allocated to each of four abundance levels. In our analyses, three species in the lowest abundance category ( *Bifidobacterium adolescentis*, *Enterococcus faecalis* and *Schaalia odontolytica*) were not assembled by either of the metagenomic assembly methods and therefore could not be assessed. The Zymobiomics Gut Microbiome Standard consists of 21 species, including two fungal species ( *Candida albicans* and *Saccharomyces cerevisiae*), staggered across eight abundance levels. Again, the three species with lowest coverage ( *Clostridium perfringens*, *Enterococcus faecalis* and *Salmonella enterica*) were not recovered during assembly.

**Figure 2.**
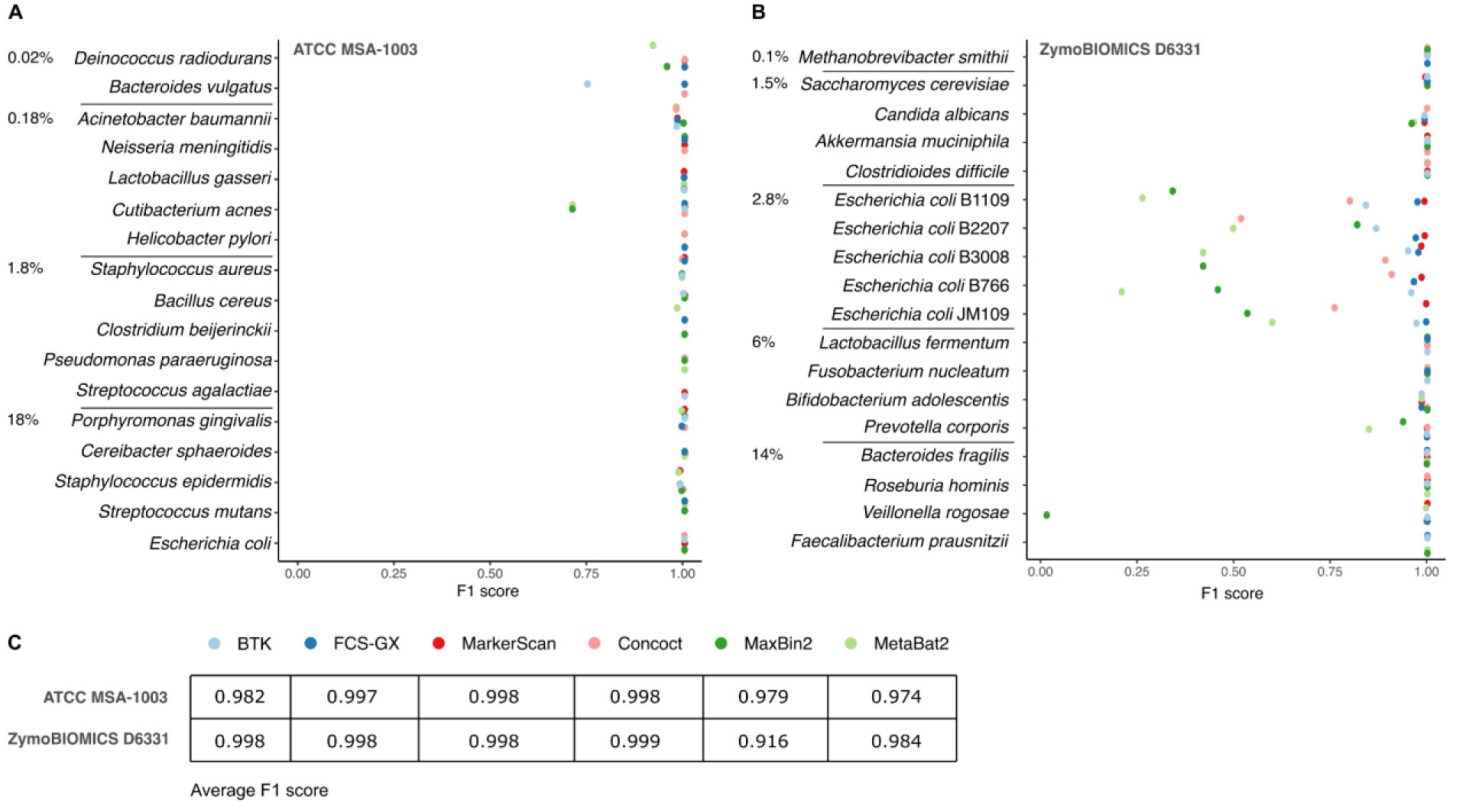
Tool comparison on classification performance on two mock communities. F1 scores of six different binning algorithms for two mock communities (ATCC MSA-1003, panel **A** and ZymoBIOMICS D6331, panel **B**) after assembly with metaFlye. Species are sorted according to their theoretical abundance of genomic DNA in the sample. Average F1 scores across both mock communities (excluding the five *E. coli* strains in ZymoBIOMICS D6331) is summarised in a table, panel **C**.

All tools achieved high accuracy and precision for the ATCC MSA-1003 community dataset assembled by metaFlye ( Figure 2A). MetaBat2 and MaxBin2 did not place the two contigs for *Cutibacterium acnes* in the same bin, with MaxBin2 placing one contig in the *Deinococcus radiodurans* bin. The binning by MetaBat2 generated a bin that was composed solely of *Deinococcus radiodurans* contigs, but did not include all contigs from the species. BTK analysis left some contigs belonging to *Bacteroides vulgatus* unbinned, as it failed to classify them to the family level. The Zymobiomics Gut Microbiome Standard ( Figure 2B) includes a hard assembly problem as it includes five different *Escherichia coli* strains that are at the same relative stoichiometry. Because of this confluence of taxonomy and effective read coverage, only a limited number of the resulting contigs could be confidently assigned to distinct strains. Binning results on this subset of the Zymobiomics dataset are therefore difficult to evaluate. Both MetaBat2 and Maxbin2 split some of the contigs belonging to *Candida albicans* into two bins, and also over-split contigs from *Prevotella corporis*. Maxbin2 did not classify the complete circular genome of *Veillonella rogosae*.

### MarkerScan put to work: five real-world examples

To demonstrate the power of MarkerScan, here we compare its performance to that of BTK and FCS-GX on three samples originating from the DToL project ^3^, one from the VGP ^53^ and one from ASG ^54^. These five datasets were selected to reflect the increasing complexity that can be observed in target species and their cobiomes in nature.

**Figure 3.**
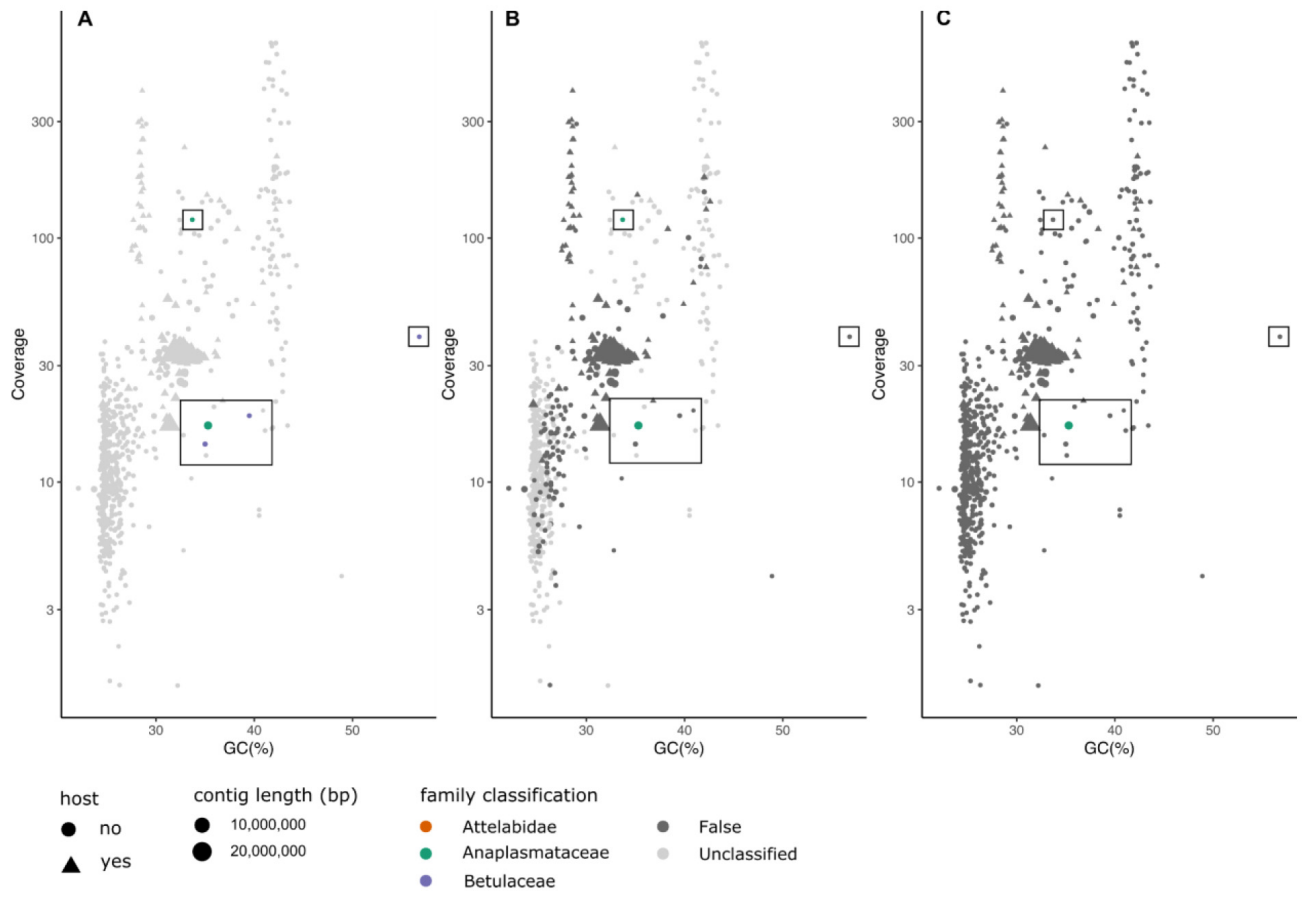
Comparison of classification results of MarkerScan ( **A**), FCS-GX ( **B**) and BlobToolKit ( **C**) of hifiasm assembly of *Apoderus coryli*. Points plotted as triangles represent contigs which align to the submitted, manually curated chromosomal assembly of the target species. The size of each point reflects the contig length. Points are coloured according to the family-level classification of each of the methods. “False” indicates family-level classifications made by the tool that are incorrect based on mapping of the contigs to the reference genome. The squares highlight contigs deriving from a *Wolbachia* endosymbiont (green) and from the hazel plant food host (mauve).

***Apoderus coryli*.** The hazel-leaf roller weevil ( *Apoderus coryli*) is a leaf-rolling beetle found in deciduous woodland in Europe and Western Asia where its main host plant, the common hazel ( *Corylus avellana*, in family Betulaceae) is present. The DToL project generated PacBio HiFi data from a single individual caught in Wytham Woods, in Oxfordshire, UK. In the primary assembly of PacBio HiFi data for this species, three short contigs that contained an SSU most closely matching common hazel were flagged by MarkerScan as deriving from the family Betulaceae ( Figure 3A). These likely derive from food ingestion. FCS-GX identified the same three contigs for removal, although they were incorrectly assigned to the walnut family (Juglandaceae) ( Figure 3). The Betulaceae contigs were not identified in BTK. The sequenced specimen was infected with the endosymbiotic bacterium *Wolbachia* (Anaplasmataceae). All three methods identified a large contig as being derived from Anaplasmataceae but another smaller fragment was only detected by MarkerScan and FCS-GX. FCS-GX did not flag this contig for removal ( Figure 3). Reassembly of reads identified as deriving from *Wolbachia* by MarkerScan resulted in the generation of an improved, complete circular *Wolbachia* genome (submitted to INSDC as OX366367.1) ( Table 3). Both FCS-GX and BTK were not successful in identifying the correct family for this weevil. MarkerScan does not aim to identify the target species and therefore host contigs were marked as unclassified ( Figure 3).

**Table 3.** Summary statistics of the assembly of the largest contaminating fraction in each sequencing dataset according to MarkerScan, FCS-GX and BTK.

Dataset	Family	Tool	Completeness *	Length (bp)	N50 (bp)
*Apoderus* * coryli*	Anaplasmataceae	MarkerScan	C:99.8%[S:99.5%,D:0.3%],F:0.0%,M:0.2%,n:364	1,643,928 linear	1,613,586
		MarkerScan reassembly	C:99.8%[S:99.5%,D:0.3%],F:0.0%,M:0.2%,n:364	1,610,943 circular	1,610,943
		FCS-GX	C:99.8%[S:99.5%,D:0.3%],F:0.0%,M:0.2%,n:364	1,643,928 linear	1,613,586
		BTK	C:99.8%[S:99.5%,D:0.3%],F:0.0%,M:0.2%,n:364	1,613,586 linear	1,613,586
*Thunnus * *albacares*	Kudoidae	MarkerScan	C:0.3%[S:0.3%,D:0.0%], F:0.1%,M:99.6%,n:954	183,935	45,507
		MarkerScan reassembly	C:5.3%[S:5.1%,D:0.2%], F:2.4%,M:92.3%,n:954	4,461,306	93,664
		FCS-GX	C:15.8%[S:15.7%,D:0.1%],F:6.4%,M:77.8%,n:954	19,962,157	75,924
		BTK	Not identified	0	0
*Mythimna * *impura*	Nosematidae	MarkerScan	C:96.4%[S:5.7%,D:90.7%],F:1.5%,M:2.1%,n:600	22,731,251	617,122
		MarkerScan reassembly	C:96.3%[S:2.8%,D:93.5%],F:1.5%,M:2.2%,n:600	26,161,197	586,769
		FCS-GX	C:96.4%[S:5.7%,D:90.7%],F:1.5%,M:2.1%,n:600	22,731,251	617,122
		BTK	C:96.4%[S:5.7%,D:90.7%],F:1.5%,M:2.1%,n:600	22,731,251	617,122
*Cladonia* * squamosa*	Trebouxiaceae	MarkerScan	C:41.2%[S:41.1%,D:0.1%],F:2.8%,M:56.0%,n:1519	21,120,226	24,677
		MarkerScan reassembly	C:87.1%[S:85.2%,D:1.9%],F:3.2%,M:9.7%,n:1519	62,606,723	1,271,318
		FCS-GX	C:41.2%[S:41.1%,D:0.1%],F:2.8%,M:56.0%,n:1519	26,044,776	24,486
		BTK	Not identified at family level	0	0

* BUSCO completeness, where C is complete, S is single copy, D is duplicated, F is fragmented, M is missing, and n is the number of loci in the BUSCO set used in the analyses.

**Figure 4.**
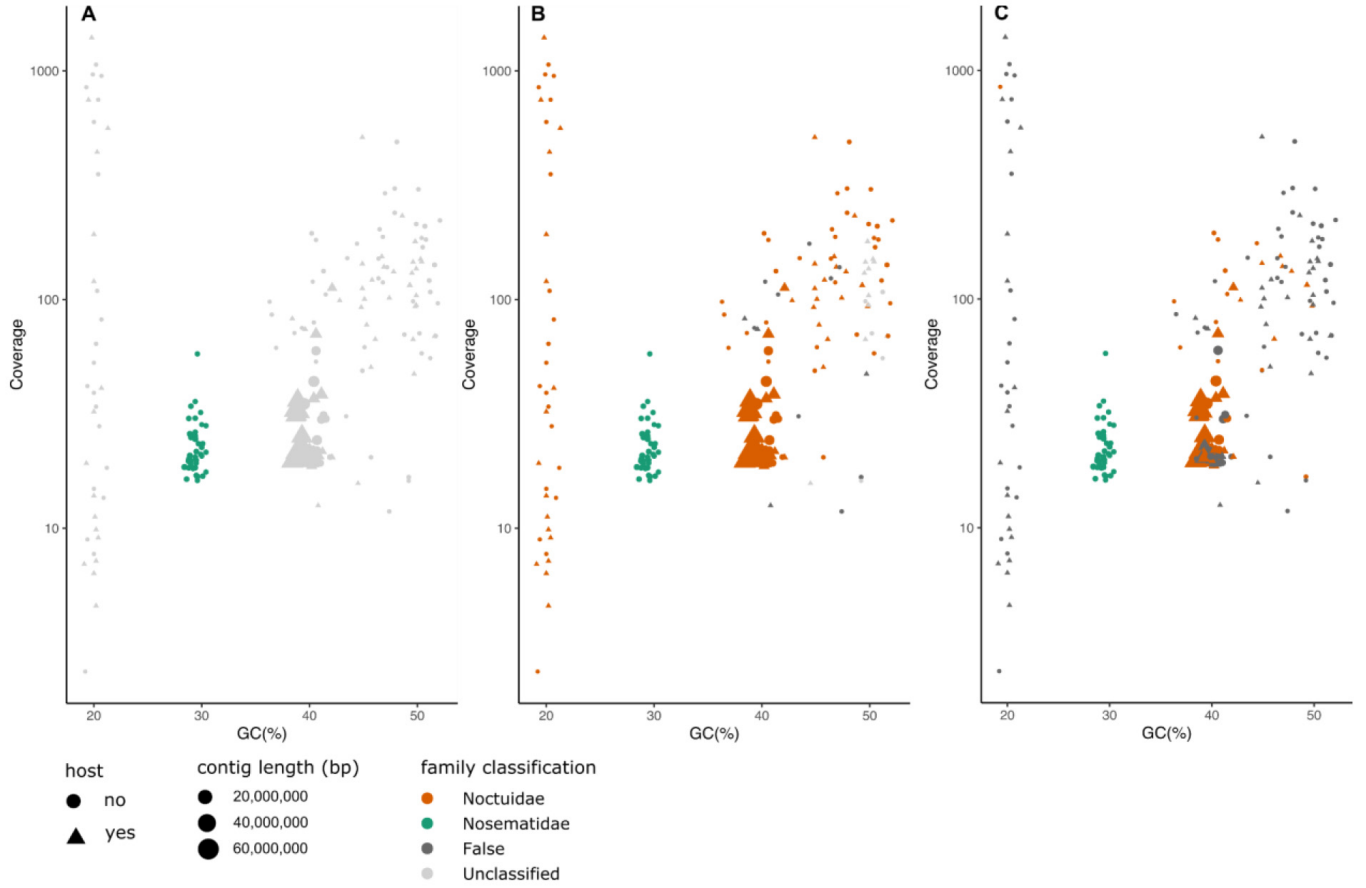
Comparison of classification results of MarkerScan ( **A**), FCS-GX ( **B**) and BlobToolKit ( **C**) of hifiasm assembly of *Mythimna impura*. Points plotted as triangles represent contigs which align to the submitted, manually curated chromosomal assembly of the target species. The size of each point reflects the contig length. Points are coloured according to the family-level classification of each of the methods. “False” indicates family-level classifications made by the tool that are incorrect, based on mapping of the contigs to the reference genome.

***Mythimna impura*.** A more complex issue arises when genomic data for a eukaryotic target species is contaminated with other eukaryotic species, whether parasites, commensals or food sources. The smoky wainscot ( *Mythimna impura*) is a nocturnal moth whose larvae feed on grasses. *M. impura* has a widespread distribution across the Palearctic, and the DToL specimen was again sourced from Wytham Woods, Oxfordshire. All three methods successfully identified the presence of a parasitic microsporidian (Nosematidae) in the sample and flagged the same 38 contigs to be separated from the host genome. These contigs had a distinct GC profile and coverage ( Figure 4). Reassembly of reads identified as microsporidian generated a genome draft with greater span, and also with higher fraction of “single copy” marker genes present in more than one copy. This likely reflects the ability of the assembler to better represent the tetraploidy of *Nosema* microsporidia ^55, 56^ ( Table 3, Figure S6).

**Figure 5.**
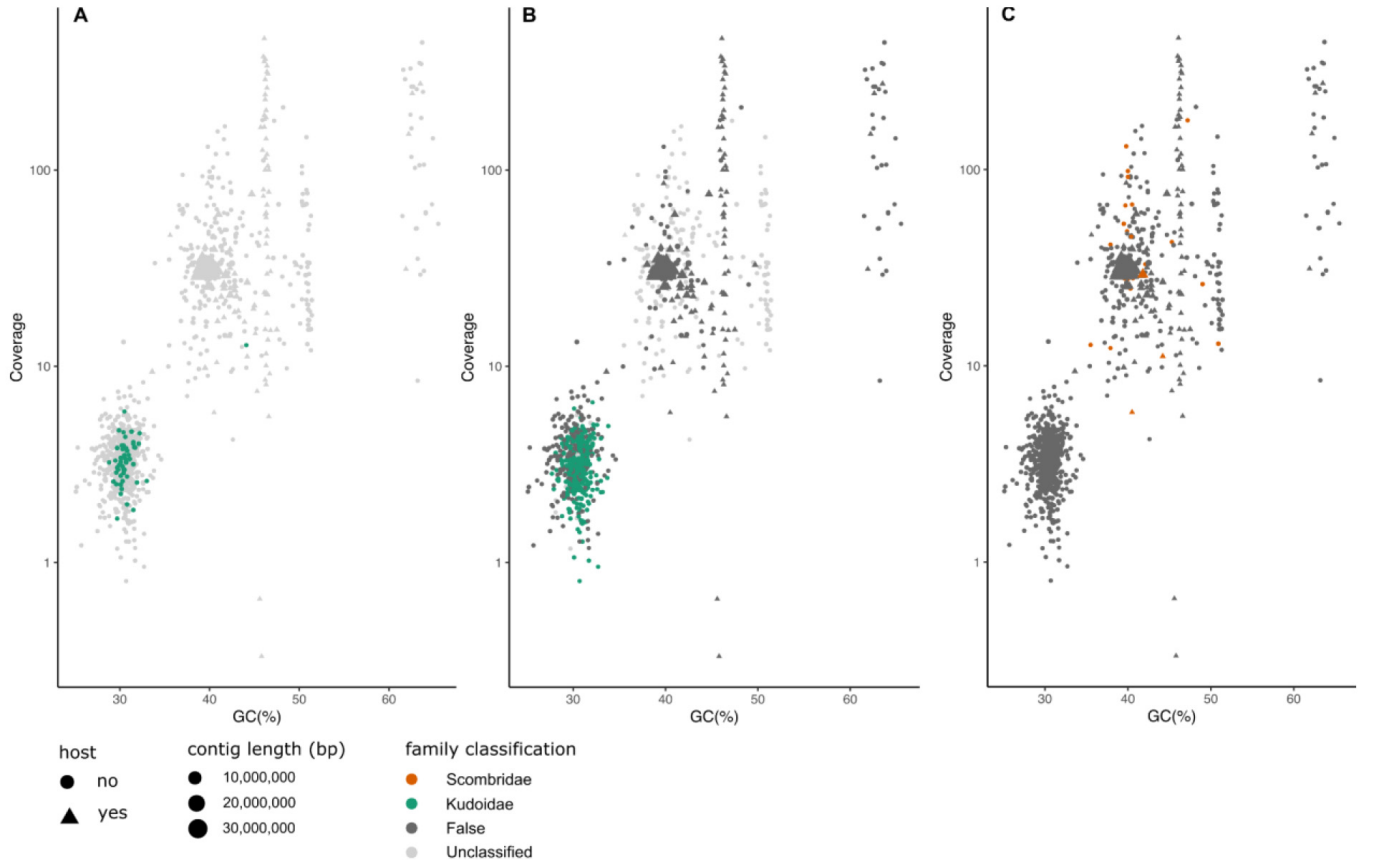
Comparison of classification results of MarkerScan ( **A**), FCS-GX ( **B**) and BlobToolKit ( **C**) of hifiasm assembly of *Thunnus albacares*. Points plotted as triangles represent contigs which align to the submitted, manually curated chromosomal assembly of the target species. The size of each point reflects the contig length. Points are coloured according to the family-level classification of each of the methods. “False” indicates family-level classifications made by the tool that are incorrect based on mapping of the contigs to the reference genome.

***Thunnus albacares*.** The yellowfin tuna ( *Thunnus albacares*) is a larger tuna species that can be found in pelagic waters of tropical and subtropical oceans worldwide. The sequenced sample was collected for the VGP from the Coral Sea off the coast of Australia, and sequenced by Tree of Life, Wellcome Sanger Institute. It was noted, after sequencing, that the sample was infected by the myxozoan *Kudoa*. *Kudoa* species parasitise the muscle tissue of marine fish and cause significant economic losses in commercial fishing. Myxozoans are obligate parasites, placed as a subphylum of Cnidaria, and are understudied. In particular they are not well-represented in public sequence databases. As all the methods we compared require public sequence for classification, we expected poorer performance. Both MarkerScan and FCS-GX identified the presence of *Kudoa* in the sample, with FCS-GX better in terms of the number of contigs correctly assigned to *Kudoa*. Despite this, none of the classified contigs were flagged for removal by FCS-GX in its final report file ( Figure 5).

***Cladonia squamosa*.** Lichens are composite organisms which consist minimally of a fungus (the mycobiont) and a photosynthetic partner (the photobiont). The photobiont can be either a eukaryotic green alga or a cyanobacterium. Metabarcoding and emerging metagenomic analyses suggest that lichens can harbour additional cobionts, with several fungi, several photobionts and a community of other organisms (largely prokaryotes) present in a single lichen holobiont. The Linnaean binomial refers to the fungus. The dragon cup lichen ( *Cladonia squamosa*) has the trebouxiophyte *Asterochloris erici* as major photobiont ^57^. All tools identified the fungal partner and the major photobiont. MarkerScan additionally detected bacteria from the families Acidobacteriaceae and Acetobacteriaceae, and data that derive from contamination with a mite (Arthropoda, Acari). FCS-GX and BTK also indicated the presence of Coxiellaceae bacteria. While BTK detected the presence of a green alga, it was not able to narrow down the source organism to the family level. Despite these differences, a high level of convergence was observed between the tools, with more than 99% and 98% of predictions by MarkerScan for Trebouxiaceae and Acidobacteriaceae respectively confirmed by FCS-GX. However, the green algal contigs flagged both by FCS-GX and MarkerScan did not represent a complete Trebouxiaceae genome. The Trebouxiaceae bin had a low overall span (26 and 21 Mb for FCS-GX and MarkerSan respectively) and poor N50s around 25 kb ( Table 3). Interestingly, reassembly of the Trebouxiaceae read set by MarkerScan improved the completeness of the assembly (according to the BUSCO chlorophyta_odb10 dataset) from 41% to 87%, and improved the assembly span and N50 to 63 MB and 1.25 Mb, respectively ( Table 3). Other lichen-forming Trebouxiales have draft genome assembly spans from 54 to 71 Mb ^58^.

**Figure 6.**
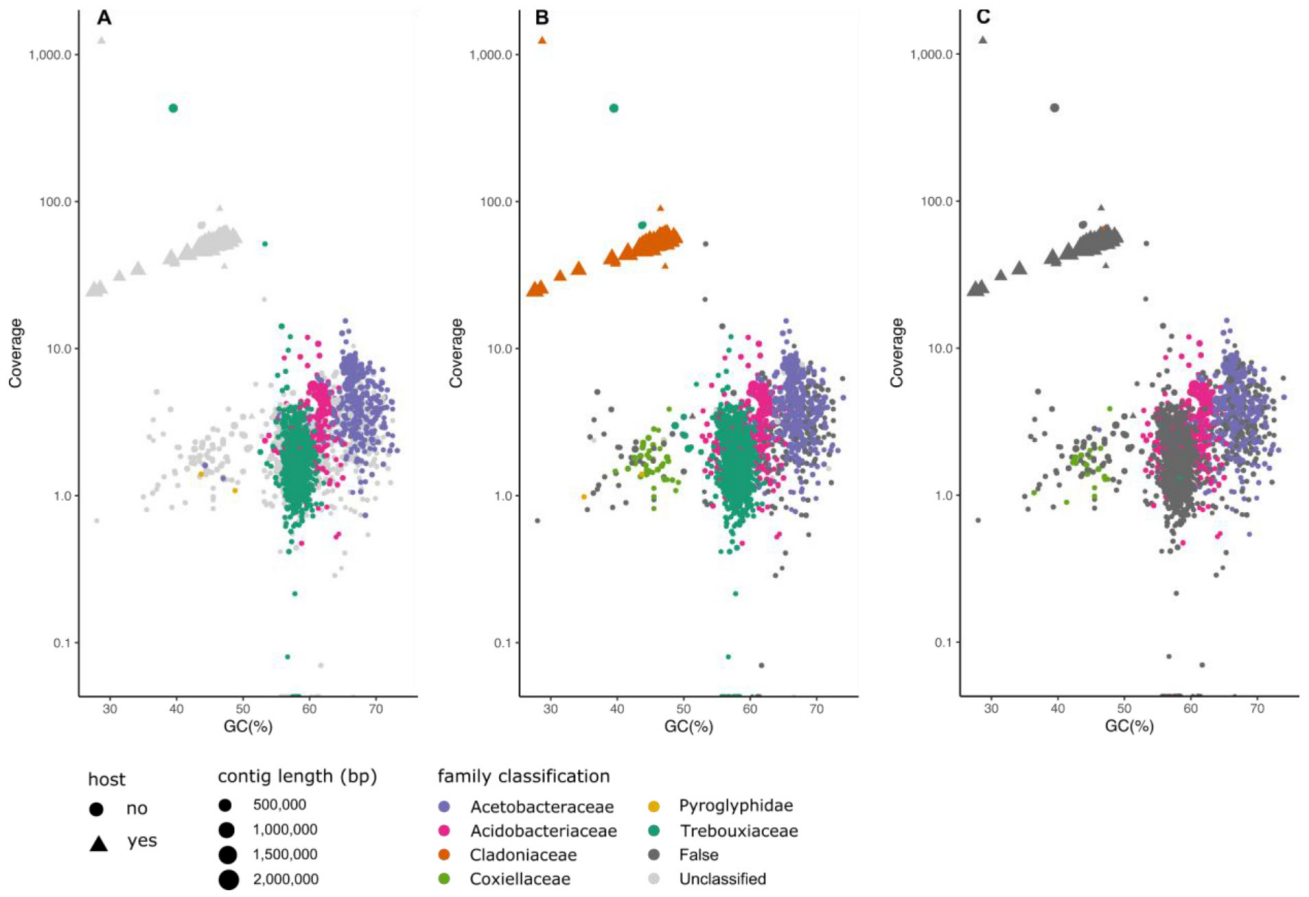
Comparison of classification results of MarkerScan ( **A**), FCS-GX ( **B**) and BlobToolKit ( **C**) of hifiasm assembly of *Cladonia squamosa*. Points plotted as triangles represent contigs which align to the submitted, manually curated chromosomal assembly of the target species. The size of each point reflects the contig length. Points are coloured according to the family-level classification of each of the methods. “False” indicates family-level classifications made by the tool that are incorrect based on mapping of the contigs to the reference genome. The “streak” of Cladoniaceae-assigned contigs in the upper left of the GC-coverage plot is typically observed in un-scaffolded fungal genome assemblies.

***Chondrosia reniformis*.** Sponges harbour dense, diverse and metastable microbial communities which can amount up to 35% of their biomass ^59^. Metagenomic approaches have been applied to disentangle the sponge host and its entire microbiome ^60^. In analysis of the *Chondrosia reniformis* PacBio HiFi dataset, MarkerScan only succeeded in detecting the families Rhodobacteraceae and Ectothiorhodospiraceae, while FCS-GX and BTK detected a wider suite of microbial partners. In the previous four examples, metagenomic binning was not particularly successful due to overpartitioning (Figure S4–7). However MetaBat2 performed well on this dataset (Figure S8).

**Figure 7.**
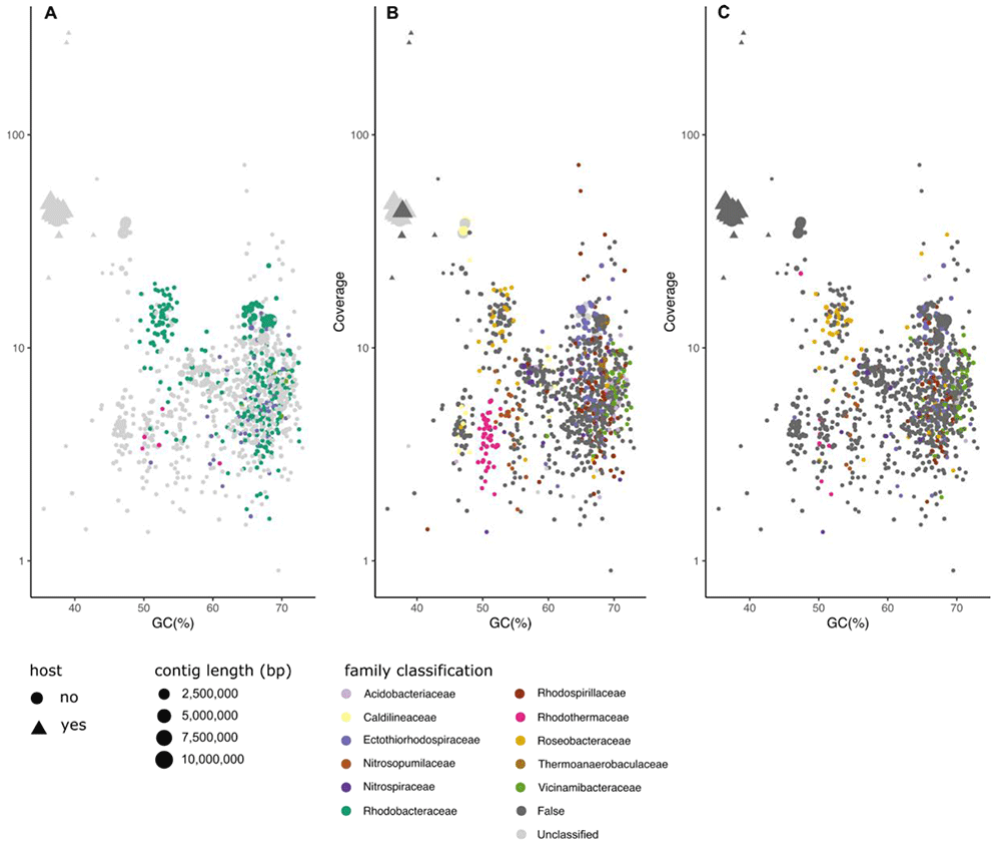
Comparison of classification results of MarkerScan ( **A**), FCS-GX ( **B**) and BlobToolKit ( **C**) of hifiasm assembly of *Chondrosia reniformis*. Triangles represent contigs which align to the submitted manually curated chromosomal assembly. Size of the shape is reflective of their size and coloured according to the family-level classification of each of the methods. “False” indicates family-level classifications made by the tool that are incorrect based on mapping of the contigs to the reference genome.

## Discussion

We present and compare a new workflow, MarkerScan, for identification of members in mixed-species genome sequencing datasets and separation of data into distinct, assembled genomes. We demonstrate its utility in separating bacterial and eukaryotic cobionts from genome datasets produced to assemble target species. MarkerScan differs from other database-dependent methods in its prioritisation of species composition determination prior to sequence similarity searches and sequence binning. This ensures that MarkerScan does not suffer from the high rate of false positive assignments that is characteristic of both FCS-GX and BTK, evident in the high proportion of dark grey dots in panels B and C of Figure 3– Figure 6. These false positives can make up significant proportions of an assembly. For example, even for the lightly-contaminated hazel-leaf roller weevil dataset, BTK and FCS predicted contigs to belong to more than 30 and 50 different phylogenetic families respectively. For such “low complexity metagenomes”, just the kinds of samples we would expect to be part of large-scale biodiversity genomics projects, MarkerScan performs very favourably. FCS-GX also had good performance for detection of all cobionts present in a sample, albeit with a relatively high false positive rate.

We identify some limitations of MarkerScan. MarkerScan relies on the presence of diagnostic SSUs in the assembled sequence set. This requirement is not always met for species in very low abundance, and data from these taxa are therefore missed by MarkerScan. Low abundance genomes will assemble poorly because of low coverage, and the completeness of such low coverage bins is usually low. We note that most genome assembly processes include curation steps that will exclude contigs of very low coverage compared to the target species assembly, and this sort of contamination will very rarely make its way into assemblies submitted to public datasets. The use of FCS-GX within the National Center for Biotechnology Information processes for accessioning assemblies to INSDC databases will also usually catch and flag for removal any remaining such sequences.

A related issue arises when analysing high species-complexity metagenomes that may contain novel prokaryotic lineages, as exemplified by the sponge dataset explored above ( Figure 7). Many common sponge-associated bacteria, such as *Poribacteria*, PAUC34f and AncK6, have not been definitively placed in bacterial taxonomies. This particularly impacts MarkerScan analyses, which relies on identifying family-level taxa from which a bespoke Kraken2 k-mer database can be built. For MarkerScan, the presence of prokaryotic species whose position in the taxonomy is poorly resolved, or of polyphyletic families, can result in inefficient identification of contigs and of reads that should be assigned to that species. We therefore would not promote the use of MarkerScan for sequencing projects where the target host is known to be associated with a diverse mix of many cobiont taxa. For these samples, established metagenomic binning tools may be more effective. Thus, while metagenomic binning was not particularly successful in the other four case studies due to overpartitioning (Figure S4–7), MetaBat2 performed well on the sponge dataset (Figure S8) highlighting the limitation of contamination detection approaches in complex community scenarios. The performance of MarkerScan will improve with increased deposition of metagenome-derived assembled genomes, and the development of a sequence similarity-based approach to prokaryotic taxonomy.

In terms of computational complexity, the high memory requirement of FCS-GX (a 400 Gb database must be available in memory) makes this tool only usable on select compute clusters. Both MarkerScan and BTK have lower memory requirements, but are characterised by markedly longer run times.

Importantly MarkerScan not only identifies the species composition of the sample, and flags sequences likely belonging to cobiont taxa, but also generates assemblies of these cobionts. We have applied this approach to detect *Wolbachia* in 368 insect samples ^61^ and succeeded in assembling 110 complete genomes. In the five case studies highlighted in this paper MarkerScan was able to improve genome assemblies for some of the cobionts (symbionts, parasites and microbiomes) present in the sample. The greatest improvement was the genome assembly of the photosynthetic partner in the lichen symbiosis ( *Cladonia squamosa*), generating an assembly more than double in size and completeness (62.6 Mb, see Table 3). The quality of this *C. squamosa* photobiont genome is now in line with released genomes of the family Trebouxiaceae (range 54–71 Mb).

MarkerScan is freely available for download and use. We will continue to improve its performance. Of particular interest is the potential for incorporation of Hi-C chromatin conformation data, often generated for the same samples by major biodiversity projects. Hi-C can be used to scaffold prokaryotic genomes from metagenomic data, and incorporation of algorithms which integrate Hi-C data such as bin3C ^62^, HiCBin ^63^ and MAGPhase ^64^ might improve performance in both binning and assembly of cobionts from these mixed-species datasets.

## Data Availability

All raw data are available in INSDC databases (see Table 2) and all results described in this paper can be accessed on Zenodo ( 10.5281/zenodo.10260141). The MarkerScan code is openly available (CCBY 4.0) from https://github.com/CobiontID/MarkerScan. Supplementary figures are available at https://doi.org/10.5281/zenodo.10640060.
