# Supplementary material for: MarkerScan: Separation and assembly of cobionts sequenced alongside target species in biodiversity genomics projects
Source: Wellcome Open Res. 2024 Feb 13;9:33. [Version 1] doi: 10.12688/wellcomeopenres.20730.1 (PMC11016177; doi:10.12688/wellcomeopenres.20730.1)
Supplement: Peer review history

# Reviewer report: Reviewer response for version 1

**DOI:** 10.21956/wellcomeopenres.22940.r76899

**Published:** 2024-04-13

**Authors:** Gertraud Burger

**Affiliations:** 1 Department of Biochemistry and Molecular Medicine, Robert-Cedergren Centre for Bioinformatics and Genomics, University of Montreal, Montreal, Québec, Canada

On a general note,  authors describing bioinformatics tools should consider to provide web assess at least to a demo version of their software, for testing by reviewers and potential users alike.

 Overall, the manuscript is interesting and clearly written. 

 Major issues

 ------------

 1. In my opinion, it's a serious shortcoming that MarkerScan doesn't verify the assumed taxonomy of the target species (p.7).

 2. MarkerScan reports contaminant bacterial species (p.9), but no evidence is provided that the 'detected bacterial sequences' are indeed in the sample.

 Minor points

 -------------

 Methods - Workflow

 - Please clarify in how far the workflow depends on the PacBio technology, and if the other long-read technologies are also supported.

 p.4 left col. 

 - "The putative SSU sequences are extracted.."  Does 'sequences' mean 'reads'? Clarify.

 - "For every observed family, all publicly available genomes". Clarify: genomes from ALL species of a given family?

 Result section:

 - The headers in the Results should more explicitly state the subsections' content. The first subsection seems to compare binning while the second deals with taxonomic assignment. Without such clarification, the selection of the tools benchmarked later appears arbitrary.

 p.7

 - Justify why MarkerScan was not compared to e.g., Concoct, which has higher F1 scores in Fig. 2C.

 - Spell out INSDC

 - "Mythimna impura. A more complex issue arises, when..contaminated..." Please rephrase, because 'eukaryotic contamination' is not specific to M. impura, but was already an issue in the weevil example.

 Fig. 3. 

 - Mauve: not visible. Also, mention in the legend the particular species that fall in the Attel., Anapl., and Betu. families

 p.11. 

 - "We identify some limitations of MarkerScan." To avoid that users waste their time and resources, it would be desirable that MarkerScan performs a pre-scan to warn if the mentioned limitations will likely affect the results.

 p.12 

 - "lower memory...". Hardware and operating system requirements of MarkerScan should be clearly stated and compared in general terms with that of the other software mentioned here.

Is the rationale for developing the new method (or application) clearly explained?

Yes

Is the description of the method technically sound?

Yes

Are the conclusions about the method and its performance adequately supported by the findings presented in the article?

Yes

If any results are presented, are all the source data underlying the results available to ensure full reproducibility?

Yes

Are sufficient details provided to allow replication of the method development and its use by others?

Yes

Reviewer Expertise:

Eukaryotic genome evolution, comparative genomics.

I confirm that I have read this submission and believe that I have an appropriate level of expertise to confirm that it is of an acceptable scientific standard, however I have significant reservations, as outlined above.

# Reviewer report: Reviewer response for version 1

**DOI:** 10.21956/wellcomeopenres.22940.r76898

**Published:** 2024-04-13

**Authors:** Timothy A Crombie

**Affiliations:** 1 Florida Institute of Technology, Melbourne, Florida, USA

**Summary**

 The manuscript presents a novel tool (MarkerScan) that identifies, separates, and assembles sequences for distinct taxa found within mixed-species genome sequencing datasets. The rationale for developing this tool is that many sequencing datasets contain numerous sequencing reads from non-target species. The contaminating sequences can arise from errors in sample preparation or through tight biological associations of taxa with a target-species. 

 To avoid the buildup of mislabeled sequences in public archives, various tools have been developed to identify and filter non-target contaminants. MarkerScan distinguishes itself from previous tools in at least two ways. First, MarkerScan uses the universal marker small subunit rRNA (SSU) to screen for contaminating taxa prior to classification. Second, rather than discarding the non-target reads, MarkerScan will produce high-quality assemblies for non-target species identified within the sequencing dataset.

 The description of the MarkerScan is thorough, clear, and direct.

 This reviewer has no issues with any step of the workflow or its description in the manuscript.  The example output data provided in the zenodo repository does not provide code to recreate the analysis, but it does give them an example of the full output of the tool for the numerous examples included in the text.

 The authors provide examples showing the utility of the MarkerScan in identifying, separating, and assembling non-target species genomes from datasets that range in complexity. The authors point out that MarkerScan’s unique order of operations (identification of species composition first, then sequence similarity search and binning) greatly reduces the high rate of false positive assignments characteristic of some other methods - especially for examples of “low complexity metagenomes”. This is key because that is exactly the kind of sequencing projects the tool is designed to work with and other tools are prone to over partitioning data. In contrast, the authors point out that the tool is less appropriate for “high-complexity metagenomes”. 

**Major concerns**

 None.

**Minor concerns**

1. The github repo appears to not align with the paper on all points e.g. “MarkerScan uses a set of reference databases, which are automatically downloaded when absent or more than one month old.” Github says 180 days? Also, the schematic overview figure in the repo is less clear than the one in the manuscript. 
2. Figure 2A+B are somewhat hard to discern because of the large number of points with F1 scores of 1.00. The authors might consider plotting these scores as a heatmap instead with tools on the x-axis and F1-scores shown on a color scale. However, the current visualization is sufficient . It could easily be the case that small differences near 1 would be indiscernible with the proposed heat map. 

**Specific notes:**

1. Introduction - paragraph2 - sentence 1: Consider changing “Although contamination can be introduced during many stages of handling of the sample…” to “Although contamination can be introduced during many stages of sample handling…”
2. Introduction - paragraph6: Consider breaking the sentence starting with “MarkerScan does this by first identifying the taxa likely to be present in a sample through…” into two or more sentences. 
3. Methods -  paragraph2 - sentence2: Consider changing “An HMM profile…” to “A profile hidden Markov model (pHMM)...”

Is the rationale for developing the new method (or application) clearly explained?

Yes

Is the description of the method technically sound?

Yes

Are the conclusions about the method and its performance adequately supported by the findings presented in the article?

Yes

If any results are presented, are all the source data underlying the results available to ensure full reproducibility?

Partly

Are sufficient details provided to allow replication of the method development and its use by others?

Yes

Reviewer Expertise:

bioinformatics, quantitative genetics, population genomics

I confirm that I have read this submission and believe that I have an appropriate level of expertise to confirm that it is of an acceptable scientific standard.

# Reviewer report: Reviewer response for version 1

**DOI:** 10.21956/wellcomeopenres.22940.r75945

**Published:** 2024-04-13

**Authors:** Laszlo G Nagy

**Affiliations:** 1 Biological Research Center, Szeged, Hungary

Vancaester and Blaxter present a new software for dealing with mixed samples in genome sequencing projects. The paper is well-written with well-placed analyses provide sufficient proof that underscores the utility of the new software. I have only small suggestions that the Authors may consider when revising their MS.

 Possibly the largest concern is that MarkerScan searches at the family level only. Fixing the search at the family level has the risk of certain taxa not having any representatives from the same family in sequence databases. While this is unlikely to be a problem in metazoans or plants, in several protist groups it may be. Is there an easy fix around? If not this might need to be acknowledged.

 In the Results section, it would be helpful to potential readers to mention the run times of MarkerScan.

 The discussion mentions higher false positive rates for FCS-GX. It may be helpful if the Authors could highlight these cases better already in the results section.

 At the cost of appearing self-promoting, I think the Authors should mention ContScout, a recent software that by far outperformed all prior contamination removal tools (Balint et al 2024 Nat. Comms). Though it works at the protein level, so may not be comparable to the tools used in this paper, it could be considered in the listing of contamination removal tools.

 On a sidenote I wonder if the Authors have discovered the irony of using a species called Mythimna impura in an analysis of genomic impurities.

Is the rationale for developing the new method (or application) clearly explained?

Yes

Is the description of the method technically sound?

Yes

Are the conclusions about the method and its performance adequately supported by the findings presented in the article?

Yes

If any results are presented, are all the source data underlying the results available to ensure full reproducibility?

Yes

Are sufficient details provided to allow replication of the method development and its use by others?

Yes

Reviewer Expertise:

bioinformatics, evolutionary genomics

I confirm that I have read this submission and believe that I have an appropriate level of expertise to confirm that it is of an acceptable scientific standard, however I have significant reservations, as outlined above.

# Reviewer report: Reviewer response for version 1

**DOI:** 10.21956/wellcomeopenres.22940.r76891

**Published:** 2024-04-09

**Authors:** Weilong Guo

**Affiliations:** 1 China Agricultural University, Beijing, China

The manuscript presents MarkerScan, a novel tool for identifying and assembling genomes of cobionts present in mixed-species genome sequencing datasets. The approach leverages the identification of small subunit rRNA (SSU) sequences to classify sequences and separate non-target genomes from the target species. MarkerScan not only provides an effective strategy for the contamination issues in public databases caused by mislabeled sequences but also facilitates the generation of high-quality genomes for non-target organisms.

 Majors:

 1. The introduction would benefit from a clearer statement of the problem being addressed. The introduction emphasized the significance of addressing the contamination issue in public databases due to mislabeled sequences and detailed the approaches that might be employed in identifying non-target sequences. However, it is essential to articulate more explicitly the challenges faced by existing tools in identifying and assembling non-target genomes within mixed-species datasets.

 2. The manuscript has indicated that the five datasets selected reflect the complexity observed in target species and their cobionts in nature. However, the selection of species primarily focuses on a relatively narrow range of ecosystems and host species. Does this selection fully represent the diversity and complexity encountered in biodiversity genomics projects? Could the choice of species from different ecological environments influence the detection performance of MarkerScan?

 3. The manuscript compares the performance of MarkerScan with other detection tools using several case analyses of mock and natural communities. However, these case studies seem somewhat fragmented, making it challenging to synthesize and understand MarkerScan’s advantages comprehensively. Incorporating systematic analyses and comparisons of MarkerScan against other tools through statistical charts could provide a more intuitive and clear depiction for readers. Furthermore, the manuscript lacks a comparative analysis of detection tools in terms of memory usage and runtime.

 4. Does prioritizing species composition determination before sequence similarity searches and binning introduce a detection bias, potentially increasing the false-negative rate? Beyond awaiting the increase of metagenome-derived assembled genomes and the development of sequence similarity-based prokaryotic taxonomy methods, are there alternative strategies for refining MarkerScan to more accurately identify species in high-complexity metagenomes?

 5. The mention in the final paragraph of the discussion regarding the potential for integrating Hi-C chromatin conformation data. The manuscript would benefit from a more detailed discussion on how Hi-C data could be used to improve performance in both binning and assembly of cobionts from mixed-species datasets.

 Minors

 1. In the final two paragraphs of the Results section, the analysis results for *Cladonia squamosa* and *Chondrosia reniformis* are described without citing Figures 6 and 7. Please include these references.

 2. In Figure 3, the small size of the points causes the mauve solid points representing ‘Betulaceae’ and the grey solid points indicating ‘False’ to appear too similar in color, making them difficult to distinguish. Please modify the colors of the points.

Is the rationale for developing the new method (or application) clearly explained?

Yes

Is the description of the method technically sound?

Partly

Are the conclusions about the method and its performance adequately supported by the findings presented in the article?

Yes

If any results are presented, are all the source data underlying the results available to ensure full reproducibility?

Yes

Are sufficient details provided to allow replication of the method development and its use by others?

Yes

Reviewer Expertise:

Plant Genomics, Bioinformatics, Wheat genetics, machine learning

I confirm that I have read this submission and believe that I have an appropriate level of expertise to confirm that it is of an acceptable scientific standard, however I have significant reservations, as outlined above.
